# Design and Properties of Novel Hydrophobic Natural Tea Saponin and Its Organogels

**DOI:** 10.3390/gels10040225

**Published:** 2024-03-26

**Authors:** Maogong Wang, Liuxin Yan, Xuying Guo, Xinwei Xing, Fengqian Liang, Chunrui Han, Liujun Liu

**Affiliations:** 1 CNPC Engineering Technology R&D Company Limited, Beijing 102206, China; wangmg_dri@cnpc.com.cn; 2 MOE Engineering Research Center of Forestry Biomass Materials and Energy, Ministry of Education, Beijing Forestry University, Beijing 100083, China; yanliuxin0505@163.com (L.Y.); guoxy@bjfu.edu.cn (X.G.); yi3210319@bjfu.edu.cn (X.X.); liangfengqian7210401@bjfu.edu.cn (F.L.); lux@bjfu.edu.cn (L.L.)

**Keywords:** tea saponin esterification, modification, organogels

## Abstract

It was first discovered that the excellent gelation ability of tea saponin can be obtained by introducing long-chain alkyl groups of dodecanoyl chloride into the glycosyl portion with direct esterification. The modified dodecanoyl chloride–tea saponin (DC-TS) was successfully synthesized and characterized with NMR, MS, and FT-IR. The tests showed that the long-chain alkyl group was successfully introduced. Combined with SEM and X-ray diffraction patterns, we found that the stable lamellar shape gels of DC-TS were formed in a variety of solvents. More interestingly, organogel was also obtained by adjusting good solvent and poor solvent as mixed solvent. It is worth noting that the driving force of organogels is the combination of hydrogen bonding and the hydrophobic interaction of the introduced alkyl chains with the rigid backbone of pentacyclic triterpenes. The modified tea saponin, a natural green surfactant, was discovered to have gelation properties, which has broadened tea saponin’s scope of application and made it more promising.

## 1. Introduction

Tea saponin (TS), a kind of oleanolane-type pentacyclic triterpenoid saponin extracted from oleaginous seeds, consists of a polymorphic mixture of glycosomes, glycosides, and organic acids [1]. It is a nonionic surfactant with emulsifying [2], foaming [3], and wetting properties [4] and widely used in food and daily products. High-purity tea saponins usually have antibacterial [5], anti-inflammatory [6], antioxidant, and other biological activities. However, the surface tension of natural TS is usually above 45 mN/m, which is relatively too high, indicating very weak surface activity. To improve its surface activity and bioactivity, some attempts have been made to modify it. 

Fan et al. [7] prepared TS-based succinic acid sulfonate, an anionic foaming agent, by esterification modifying TS with maleic anhydride (MAH) and then by sulfonation reacting with sodium bisulfite. Li et al. synthesized a novel graphene-based material of tea saponin-functionalized reduced graphene oxide (TS-RGO) via a facile thermal method. Under appropriate conditions, the TS-RGO was considered to be a cost-effective and promising material for the removal of Cd(II) from wastewater [8]. Marciani, D. modified the 3-glucuronic acid (GlcA) residue from the Quillaja saponin (QS) adjuvants by N-acylation, which yields derivatives with linear alkylamides that show structural and functional changes [9]. Huo et al. used the ability of oleanolic acid to act as an aglycone for glycosylation to design a pesticide. The activity of the synthesized glycosides against *M. oryzae* was assessed in vitro, based on the mycelium growth rate, and all showed different activities against *M. oryzae* according to their different structures [10]. The application of tea saponin modification has appeared in many fields, but there is no report related to the direct preparation of organogels from modified tea saponin. Feng’s modification scheme of directly esterifying modified tea saponin to introduce additional alkyl chains into tea saponin in the oligosaccharide part inspired us [11]. The formation of small-molecule organogels relies on non-covalent bonds such as hydrogen bonding, van der Waals interactions, coordination, π-π stacking, electrostatic interactions, and hydrophobic interactions, which self-assemble into a variety of ordered aggregates such as vesicles, micelles, nanotubes, and helical bands under non-covalent bonding [12]. Tea saponin contains a large number of hydroxyl groups and is highly hydrophilic and cannot self-assemble into ordered structures in organic solvents. The introduction of alkyl chains enhances the hydrophobic ability of tea saponin to become amphiphilic molecules, which occupy an important position in the field of self-assembly [13], providing the possibility [14] for the preparation of organogels from modified tea saponin.

## 2. Results and Discussion

### 2.1. DC-TS Structure Analysis

The functional groups in the TS that can react with dodecyl chloride include hydroxyl groups on sugar and carboxylic acid. Esters and anhydrides will be generated, respectively.

**Figure 1:**
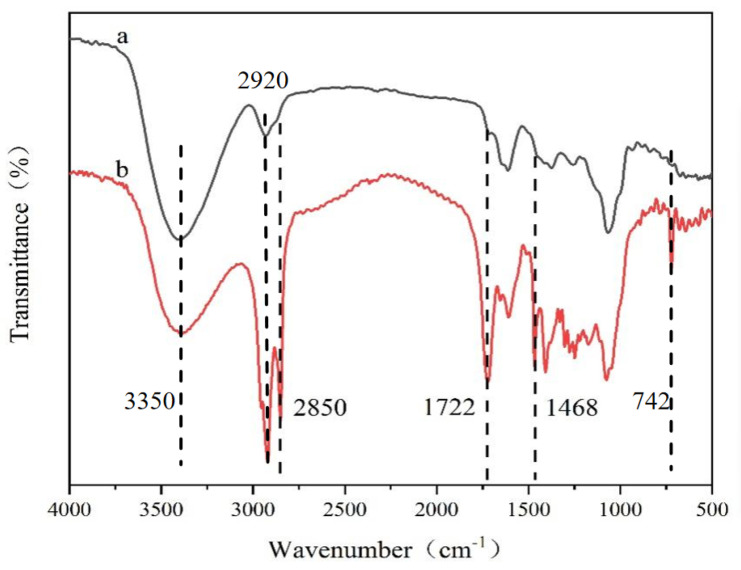
FT-IR of tea saponin and DC-TS. (a) Tea saponin, (b) DC-TS.

The FT-IR absorption spectra of TS and DC-TS are shown in Figure 1. The strong absorption peak of TS at 3350 cm^−1^ is due to the presence of a large number of hydroxyl groups on the oligosaccharide, the sharp peak at 2920 cm^−1^ is the stretching vibration of C-H in TS, and the weak peak at 1605 cm^−1^ corresponds to the C=C vibration. The values of 1405 cm^−1^ and 1080 cm^−1^ are the deformation vibrations of C-H_2_ [15]. After modification, DC-TS produced a sharp absorption peak at 2850 cm^−1^, which is a symmetric stretching vibration peak of C-H_2_, and a large number of alkyl chains were introduced into TS on the surface. The value of 1722 cm^−1^ shows a sharp vibration peak of C=O, the carbonyl group of an ester rather than an anhydride. The value of 1468 cm^−1^ shows a vibration peak of C-O-C. The appearance of these two characteristic peaks indicates the formation of an ester group. Due to the steric hindrance of the six-membered ring of sugar molecules, the methyl hydroxyl group on the sugar is more easily able to react with acyl chloride. The weak absorption peak appearing at 742 cm^−1^ is a characteristic peak of C-Cl, which may be related to the large amount of acyl chloride in the raw material and insufficient purity of DC-TS. The result showed that the dodecyl group is successfully introduced into TS through the synthetic route of Figure 1 [11].

**Figure 2:**
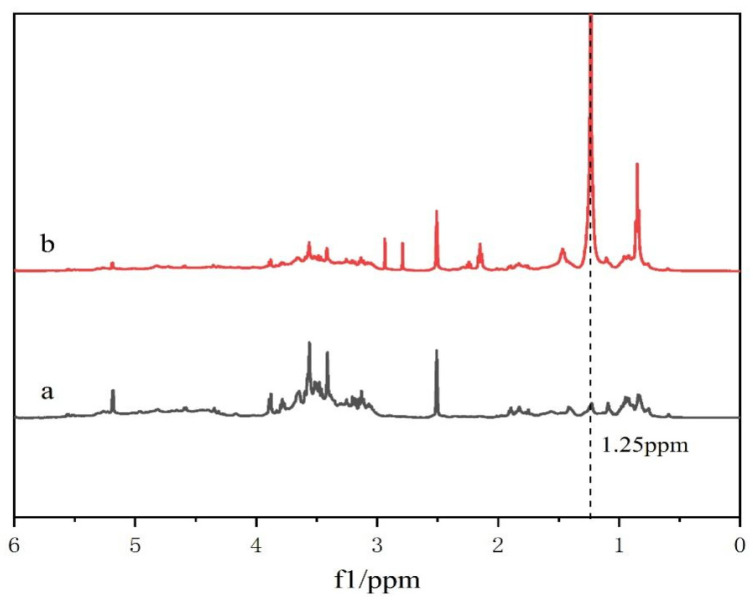
^1^H-NMR of tea saponin and DC-TS in dimethyl sulfoxide-d6. (a) Tea saponin, (b) DC-TS.

Tea saponins are composed of three parts: saponin elements, glycosomes, and organic acids. Their elements have eight different parent nucleus structures A–F and Camelliagnin B and R1-barrigenol [16], on which different glycosomes and organic acids are connected. Glycosomes are mainly composed of oligosaccharides, such as glucose, glucuronic acid, xylose, arabinose, and galactose, and organic acids mainly include angelic acid, acetic acid, tiglic acid, cinnamic acid, etc. [17], forming a complex structure of nearly one hundred structures. Therefore, ^1^H-NMR cannot accurately and appropriately represent their spectra. However, the comparison of the amplitude of the characteristic peak vibrations can be used as a further aid to analyze whether the compound is synthesized or not. As shown in Figure 2, with DMSO-d6 as the solvent, the chemical shifts of TS at 2.68–5.27 ppm mainly represent the glycosyl portion of TS, while the chemical shifts at 0.70–2.34 ppm mainly correspond to the protons of TS glycosides [18]. Contrasting the lines a and b, it can be found that -CH_2_ at the position of 1.25 ppm produces a strong and sharp peak due to the introduction of the alkyl chain. Consequently, it can be proved that dodecanoyl chloride was successfully introduced to TS.

### 2.2. Gelation Studies

**Figure 3:**
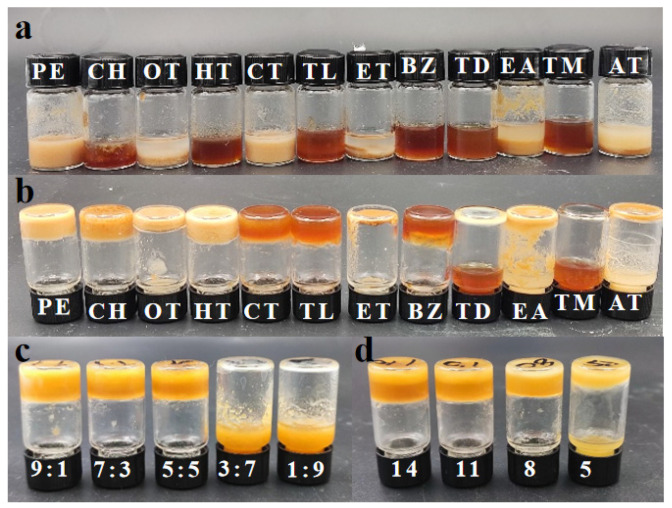
Solubility and gel capacity of DC-TS in different solvents. (**a**) Solubility of DC-TS in different solvents; (**b**) the gelation ability of DC-TS in different solvents; (**c**) the gelation ability of DC-TS in different volume ratios of cyclohexane–trichloromethane solvent mixture; (**d**) minimum gel-formation concentration (wt%) of DC-TS in cyclohexane–trichloromethane solvent mixture.

To test the gelation ability and expanding application systems of DC-TS, 12 solvents such as alkanes, halogenated hydrocarbons, aromatics, ethers, and esters were used. The gelation ability was tested according to 15 wt% addition. After heating and ultrasonication, DC-TS was able to form gels in a variety of solvents, and stable gels were formed in petroleum ether, cyclohexane, n-heptane, carbon tetrachloride, toluene, and benzene, but with a different solubility and gelation ability in different solvents. As shown in Figure 3a,b, DC-TS has the best solubility in trichloromethane after heating and sonication, but the gelation effect is poor, and it still cannot form gel after being stationary. The gel formation rate in cyclohexane was the fastest, and the gel was formed after heating and ultrasonication, but the solubility was poor, and some samples were not completely dissolved. 

Therefore, the mixture of cyclohexane–trichloromethane was chosen as the solvent to have a stronger gelation ability with guaranteed solubility. The optimal ratio of DC-TS in the mixture of the cyclohexane–trichloromethane solvent and its minimum gel concentration were further analyzed. The gelation ability was studied at a concentration of 15 wt% in the volume ratios 9:1, 7:3, 5:5, 3:7, and 1:9 of cyclohexane–trichloromethane solvent mixture. It was found that DC-TS was able to form stable gels in the mixed solvent when the proportion of cyclohexane was greater than 50%, as shown in Figure 3c. After observing the gel-formation process, we found that the gelation speed is the fastest and the gel stability is the strongest at the volume ratio of 7:3. In order to continue to investigate the minimum gel-forming concentration of DC-TS in the mixed solvent, a gel gradient addition test was carried out at the volume ratio of 7:3 cyclohexane–trichloromethane. The samples were added to the mixed solvent at the concentrations of 5, 8, 11, and 14 wt%, and were heated and ultrasonicated and left to stand for a period of time, as shown in Figure 3d. When the added amount was above the concentration of 8 wt%, the gel could be formed after a period of resting, but the gel stability was poor at the concentration of 8 wt% and after violent vibration, some of them would change to the sol state. When the concentration was greater than 11 wt%, DC-TS formed a stable gel, which was maintained in a stable gel state, with excellent gel stability. Thus, the tea saponin-modified preparation of DC-TS was used as a gel agent.

The formation of organogels is the result of the synergistic driving of gelators–gelators and gelators–solvents. The Kamlet–Taft model explains the effect of solvents on the balance of the driving forces of the gel system and gel capacity in terms of the polarity parameter (π*), ability to accept hydrogen bonding (β), and ability to give hydrogen bonding (α), as shown in Table 1. Due to the contribution ability of DC-TS to hydrogen bonding (α) being almost 0, the hydrogen bond contribution ability of the solvent plays a huge role in the formation of organogels. Analysis reveals the supply of hydrogen bonds (α), and the larger the value is, the worse the organogel effect is. From this, we can know the hydrogen bond supply of solvent (α) will compete with the hydrogen bond interaction between gelators; when the hydrogen bond supply of solvent (α) is too large, the formation of organogels will be hindered. At the same time, the polarity of solvent (π*) will also affect the gel effect to some extent. The interaction between the polar solvent and gelators competes with the interaction of gelators themselves, leading to different gel effects. The Kamlet–Taft model illustrates that the solvent action is an important part of the gelation process, which is involved in the equilibrium of the driving forces of the system and indirectly affects the gel capacity.

### 2.3. Morphological Characterization

**Figure 4:**
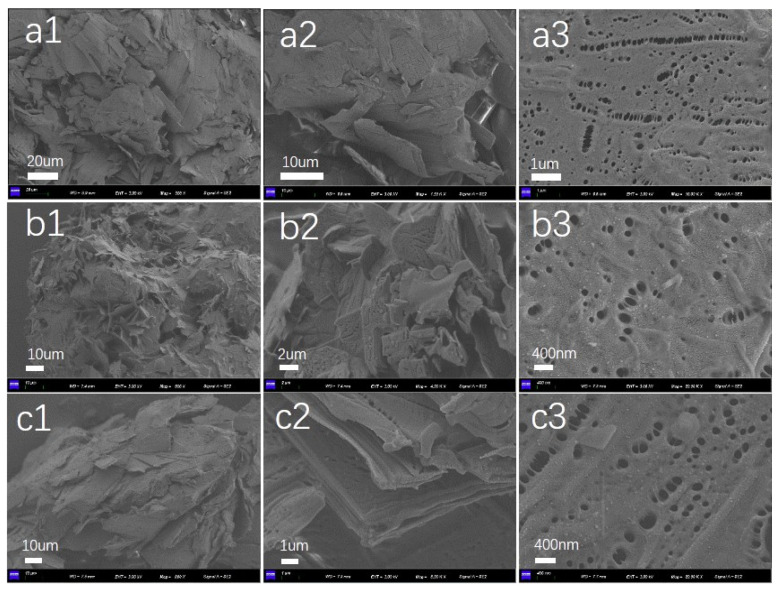
SEM images of DC-TS in organic solvent. (**a**) DC-TS in n-octane; (**b**) DC-TS in cyclohexane; (**c**) DC-TS in toluene.

In order to study the formation mechanism of organogels more conveniently, we chose three single solvent gels to observe the morphology by taking dry gels and laying them flat on slides and testing them after gold-spraying on their surfaces. As shown in Figure 4, the gels formed in n-octane, cyclohexane, and toluene were composed of irregular lamellar structures with a disordered growth direction and stacked on top of each other. The thickness and density of the lamellae are affected by the solvents [19], as shown in Figure 4b. The gel-forming ability of cyclohexane is stronger, so its lamellae are denser, and the thickness of the lamellae is about 200–500 nm. The lamellae are in turn composed of stacked smaller thin layers, as shown in Figure 4c2, but the thickness of the lamellae cannot be accurately estimated due to the tight stacking. The thickness can subsequently be further inferred by other analytical methods. Meanwhile, closely spaced elliptical pores were found on the surface of the lamellar structures of the gels formed by the different solvents, which might be the organic solvents being wrapped in the lamellar structures and the exhaust holes formed during the volatilization process.

### 2.4. Rheological Studies

**Figure 5:**
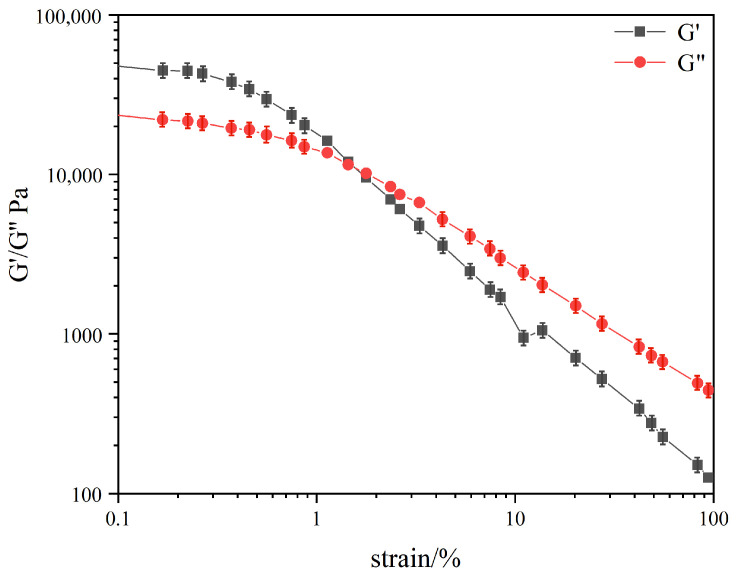
Variation in viscous modulus G″ and elastic modulus G′ of DC-TS gels in cyclohexane–trichloromethane (7:3) solvent mixture with strain.

The rheological properties of gels were extremely important for the practical application of gels, and different gel structures had a significant impact on the rheological properties [20]. In order to explore the effect of the compound spatial structure on the mechanical properties of gels, the organogel formed by DC-TS in the mixture of the cyclohexane and trichloromethane (7:3) solvents was investigated to examine the rheological properties of this system according to gelation studies. In order not to destroy the structure of the gel, a suitable strain value should be selected within the linear viscoelastic region. The relationship between the elastic modulus G′ and viscous modulus G″ of the organogel with the change in the strain value in the presence of 15 wt% DC-TS, keeping the frequency fixed, is shown in Figure 5. When the strain value was between 0.1% and 0.5%, the microstructure of the gel system remained essentially unchanged and exhibited an elastic state. When the strain value increased to 0.5%, the elastic modulus and the viscous modulus of the gel system decreased rapidly, indicating that the microstructure of the organogel started to be destroyed. When the strain value was about 1.1%, the elastic modulus G′ and the viscous modulus G″ produced the intersection point, which indicated that the gel network was completely destroyed and presented a viscous flow state. Therefore, the appropriate strain value for the DC-TS organogels was 0.5%.

**Figure 6:**
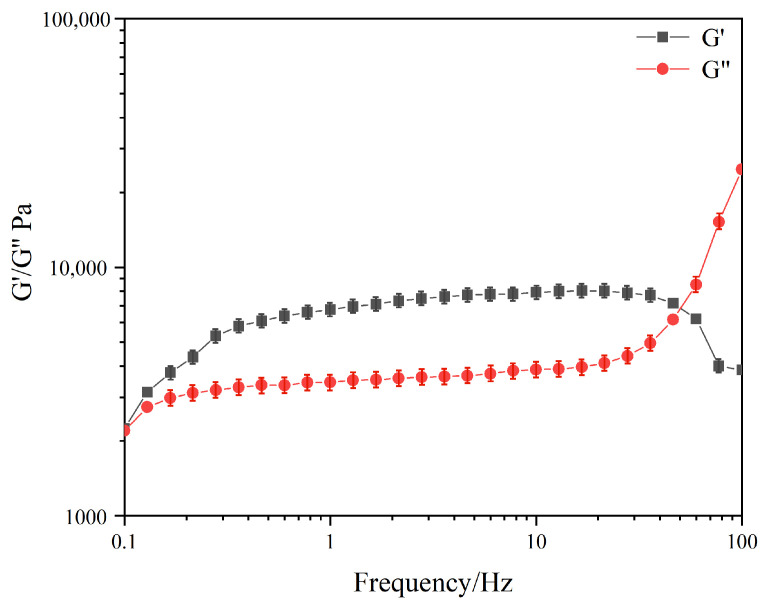
Variation in viscous modulus G″ and elastic modulus G′ of DC-TS gels in cyclohexane–trichloromethane (7:3) solvent mixture with shear frequency.

After selecting the appropriate strain, the test of the elastic modulus G′, the viscous modulus G″, and the variation with shear frequency of the DC-TS organogel was started using the oscillation mode [21]. As shown in Figure 6, in the 0.1–50 Hz range of shear frequency [21,22], the modulus of the gel system did not change significantly with the change in the shear frequency, and the elastic modulus G′ of the samples were all larger than their viscous modulus G″, showing typical elastic fluid properties with elastic modulus values of about 7800 Pa and viscous modulus values of about 3700 Pa. When the shear frequency reached 50 Hz, the intersection of the elastic modulus G′ and the viscous modulus G″ occurred, the elastic modulus G′ was smaller than its viscous modulus G″, and the organogel network was disrupted and changed from an elastomeric to a viscous flow state. It was seen that the concentration of 15 wt% of DC-TS gelling agent made the cyclohexane–trichloromethane mixed solvent system produce a stable gel system, the laminar stacked structure formed by the aggregation of the gel system had a strong mechanical strength, and the elastic modulus G′ was greater than its viscous modulus G″ in accordance with the significant characteristics of the gel. Compared with hydrogels, the G′ and G″ of small-molecule organogels of natural products are close to each other [22,23], which may be because the driving forces of the formed gels are weak, and the elasticity of the gel is insufficient.

### 2.5. X-ray Diffraction (XRD) Studies

**Figure 7:**
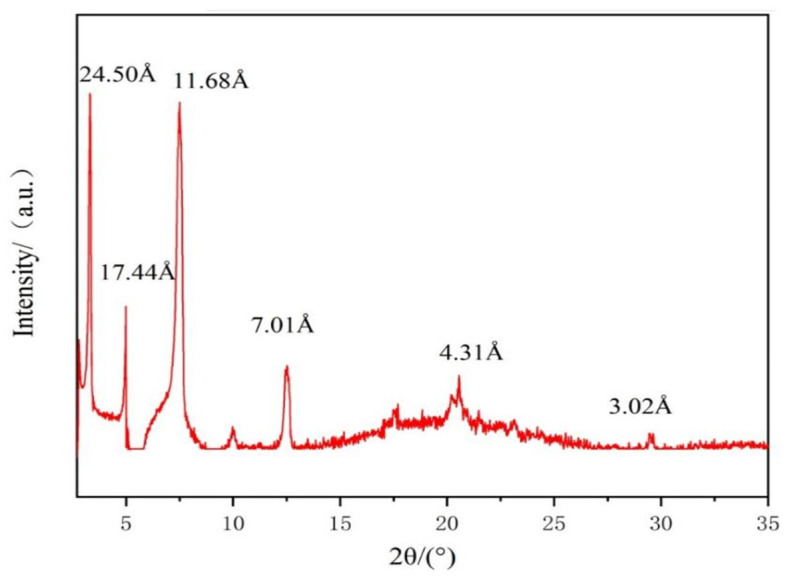
XRD patterns of xerogel of DC-TS in cyclohexane–trichloromethane (7:3) organic solvent.

The gelation mechanism of physical gels formed by relying on non-covalent bonds is different from that of chemical gels. Physical gels are formed mainly by the self-assembly of the gelling agent, and the ordered structure formed by self-assembly can be revealed by XRD [24]. In order to have a deep understanding of the supramolecular arrangement of DC-TS in the organic solution, XRD analysis of the dry gels formed in the cyclohexane–trichloromethane mixed solvent was carried out, as shown in Figure 7. The XRD diffraction pattern has many sharp diffraction angle characteristic peaks, and the crystal plane spacing was calculated by Bragg’s formula: 2dsinθ = nλ, which are 24.50 Å, 17.44 Å, 11.68 Å 7.01 Å, 4.31 Å, and 3.02 Å, which are consistent with the ratio of 1:3/4:1/2:1/4:3/16:1/8 and satisfy the lamellar structure relationship [25]. It is inferred that the dry gel formed in the cyclohexane–trichloromethane solvent mixture has a lamellar structure with a layer spacing of about 24.50 Å. The diffraction peak of 11.68 Å, as the second-strongest peak, can prove that there are more aggregates of this size, which will be attributed to the spacing of thin-layer aggregates, and multiple thin-layer structures are in turn stacked to form a lamellar structure of 200–500 nm thickness.

### 2.6. Investigation of the Driving Forces

**Figure 8:**
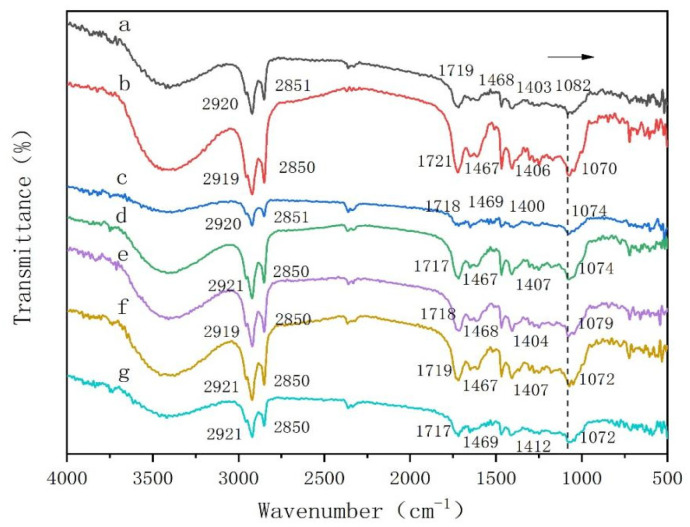
FT-IR plots of gels formed by DC-TS with different organic solvents. (a): Modified saponin powder; (b): petroleum ether; (c): cyclohexane; (d): N-heptane; (e): carbon tetrachloride; (f): toluene; (g): benzene.

Driving forces other than the hydrophobic effect of solvents during self-assembly were investigated by FT-IR and ^1^H-NMR. FT-IR of the gels was formed by DC-TS with the different organic solvents. In order to explore the main driving force for the formation of gels [26], an FI-IR test was conducted after the gel was air-dried, and the results are shown in Figure 8 and FT-IR vibration peaks of the dry gel formed by DC-TS in different solvents are shown in Table 2. By comparing the characteristic absorption peaks of the infrared spectra of gels formed with the different solvents and the modified saponin powder, the overall spectra of the gels and the modified saponin powder changed less. But the special absorption peaks after gel formation at a 2920 cm^−1^ and 2850 cm^−1^ stretching vibration of CH_2_, stretching vibration of C=O at 1719 cm^−1^, and stretching vibration of C-O-C at 1468 cm^−1^ were slightly shifted in amplitude, while the symmetric deformation vibration of CH_2_ at 1082 cm^−1^ shifted more toward the lower wave number, and the change in the position of the absorption peaks indicated that hydrogen bonding was one of the main driving forces for the self-assembly of the gel factor [27]. 

**Figure 9:**
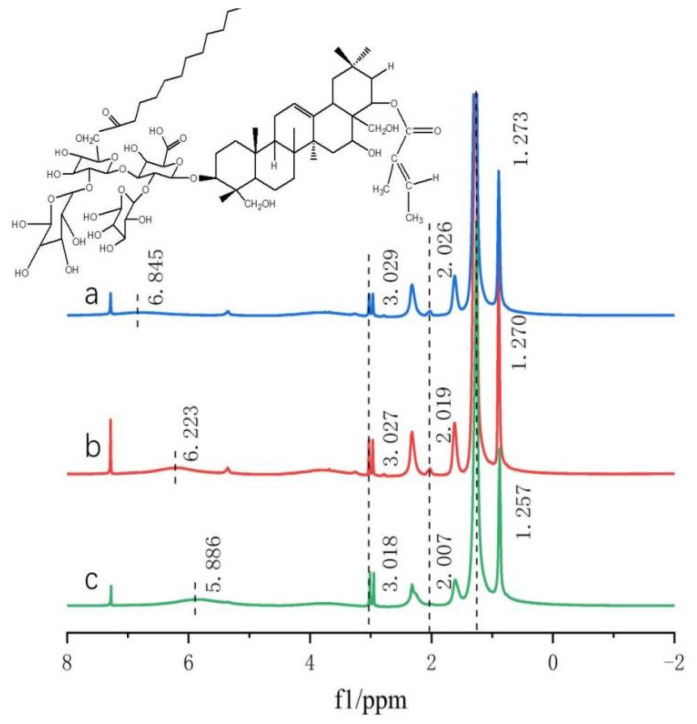
^1^H-NMR map of DC-TS in cyclohexane–trichloromethane mixture with variable concentrations. (a) DC-TS concentration of 5 wt%, (b) DC-TS concentration of 10 wt%, (c) DC-TS concentration of 15 wt%.

Concentration dependent ^1^H-NMR spectroscopy studies. The aggregation behavior of DC-TS in the mixed cyclohexane–trichloromethane solutions was studied by ^1^H-NMR. The cyclohexane–trichloromethane solutions of DC-TS at concentrations of 5, 10, and 15 wt% were tested at 25 °C. As shown in Figure 9, the chemical shift signal (Δδ) of the hydrogen proton at the organic acid position of DC-TS was continuously shifted from 6.845 ppm to 6.223, and 5.886 at the lower position when the concentration of DC-TS was increasing. The dashed lines are designed to perfectly compare peaks. At the positions of 3.029 ppm and 2.026 ppm, the hydrogen protons located on the glycosyl portion and hydroxyl group were also progressively shifted to the lower position as the concentration increased. The same result occurred for the hydrogen protons on the long-chain alkyl groups reflected at 1.273 ppm, shifting to 1.270 and 1.257. However, in the range of 5 wt% to 10 wt%, the chemical shifts were relatively small except for the hydrogen protons at the organic acid positions, which were within 10 ppm at all other positions, and larger at each position in the 10 wt% to 15 wt% range. That was consistent with the phenomenon in the previous gelation tests that DC-TS had a poor gelation at 11 wt% concentration, and a improved gelation with the increasing concentration. It was proved that the hydrogen bonding force was one of the main driving forces for gel formation [28]. Since DC-TS did not rely on the π-π stacking of amide groups and benzene rings [29], it relied on the hydrophobic effect of introducing alkyl chains and rigid skeletons of pentacyclic triterpenes and the hydrogen bonding force of hydroxyl groups to form gels. Its intermolecular non-covalent bonding force was relatively weak compared with other gelling agents, but it was proved through this experiment that it still formed stable gels under certain conditions only through the hydrophobic and hydrogen bonding forces.

### 2.7. Self-Assembly Process of DC-TS in Organic Solvents

**Figure 10:**
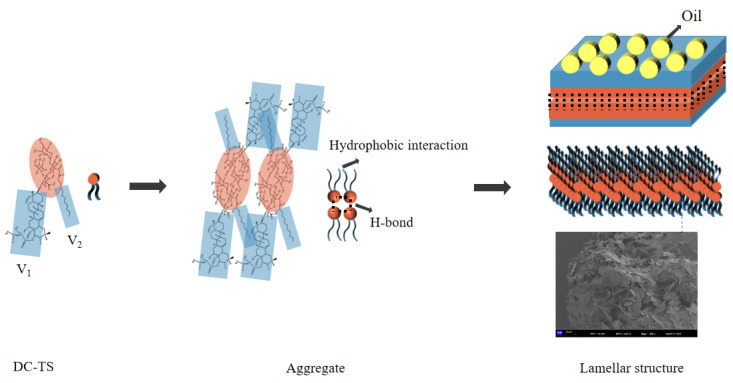
Self-assembly process of DC-TS in cyclohexane–trichloromethane solvent mixture.

Amphiphilic molecules contain both hydrophilic and lipophilic groups. When the surfactant is in aqueous solution, the lipophilic group always tends to detach from the polar aqueous environment. At low concentrations, the surfactant can spontaneously adsorb at the gas–liquid interface to form an oriented monomolecular layer. When the concentration of the surfactant continues to increase and the interfacial adsorption reaches saturation, the lipophilic group of the surfactant will spontaneously form an aggregate in aqueous solution with the hydrophilic group toward water and the lipophilic group in the interior in order to get out of the aqueous environment as much as possible, and this process is called self-assembly [30]. The balance of hydrophobic and hydrophilic forces plays a significantly important role in the self-assembly process of amphiphilic molecules in liquids, which may produce different morphologies. The formation of different morphologies is explained according to the stacking parameter P = V/al, where V and l are the volume and length of the hydrophobic part and a is the cross-sectional area of the amphiphilic hydrophilic part. When P is less than 1/3, the aggregates tend to be spherical micelles; when 1/3 < P < 1/2, they tend to form worm-like micelles; when 1/2 < P < 1, they can form vesicle structures; and when P is around 1, the aggregates are likely to be lamellar structures, and usually the stacking parameter P < 1/2 for surfactant molecules with single hydrophobic chains, while when the surfactant molecules contain two hydrophobic chains, the stacking parameter P is usually between 1/2 and 1 [31]. As shown in Figure 10, the glycosyl portion of the DC-TS molecule contains a large number of hydroxyl hydrophilic groups and also contains a hydrophobic triterpene backbone and long-chain alkyl structure. Hydrogen bonding of hydroxyl and carboxyl groups and hydrophobic interactions of triterpenoid backbone and alkyl chains may play an important role in the self-assembly of the molecule [32]. A cross-sectional area of hydrophilic groups of DC-TS is about 56.27 Å^2^ and the length of hydrophobic chains is about 13.75 Å. The volume V_1_ and V_2_ are 744.4 Å^3^ and 94.0 Å^3^, respectively. According to the stacking parameter P = V/al, P is about 1.08, which is consistent with the stacking constant of the lamellar structure. The self-assembly process of DC-TS in solvent is roughly shown in Figure 10. The gel factor DC-TS is approached to form a thin-layer structure by intermolecular hydrogen bonding and hydrophobic chain interactions. Then, in the presence of the solvent, the thin-layer structure aggregates to form a multilayer aggregated lamellar structure, and then further stacks to form a three-dimensional stacked lamellar structure, thus inhibiting the flow of liquid components in the system and forming a stable gel system.

## 3. Conclusions

In conclusion, DC-TS was synthesized from oleaginous saponin. It was shown experimentally that DC-TS has the excellent ability of gelation and forms gels in the various organic solvents including petroleum ether, cyclohexane, n-heptane, carbon tetrachloride, toluene, and benzene. Especially in the mixed cyclohexane–trichloromethane solvent, the gelator had good solubility and fast gelation above the concentration of 8 wt%. In the mixed cyclohexane–trichloromethane system, the driving forces that drive the formation of DC-TS gels were demonstrated. During the self-assembly process, DC-TS molecules formed the thin lamellar aggregates by relying on the hydrogen bonding with non-covalent bonding weak forces and hydrophobic interactions, not the π-π stacking, and then layer by layer into the lamellar stacking structures. The DC-TS synthesis method is simple, inexpensive, and has wide application prospects in industrial fields. This study shows that DC-TS has a strong van der Waals weak force and can be used to construct functional materials [33] with gel phenomenon in organic solvents. It could be applied to improve the gel ability of a long-chain petroleum system, such as the antisettling application of oil-based drilling fluid.

## 4. Materials and Methods

### 4.1. Materials

Tea saponin (purity, 98%), dodecanoyl chloride, potassium carbonate, dimethyl sulfoxide, N,N-dimethylformamide were purchased from Shanghai Macklin Biochemical Technology Co. (Shanghai, China). All chemicals were of analytical grade and used at the time of purchase.

### 4.2. Preparation of Dodecyl Chloride–Tea Saponin (DC-TS)

**Figure 11:**
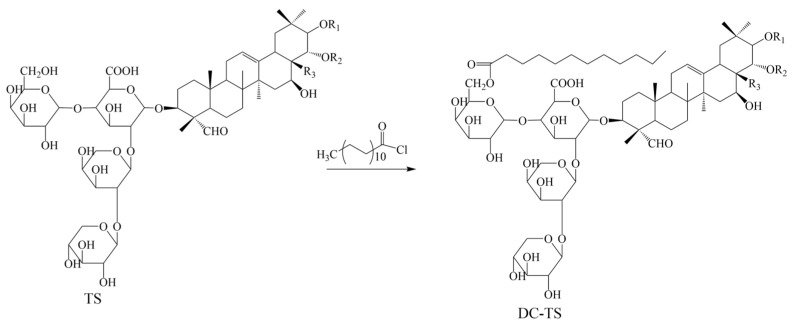
Synthetic route of DC-TS.

Based on the strong practicality of the excellent surfactant sodium dodecyl sulfonate, the dodecyl oleophilic group was introduced into tea saponin. The synthetic route of DC-TS is shown in Figure 11. The raw material ratio was designed according to the reference literature [11].

Tea saponin (360 mg, 0.3 mmol) was dissolved in 10 mL of N,N-dimethylformamide and 1.8 mmol dodecanoyl chloride and 3.6 mmol anhydrous potassium carbonate were added. The reaction was stirred at room temperature for four hours. The reaction mixture was diluted with the appropriate amount of water and then 50 g of D-101 macroporous sorbent resin was added for 1 h. The macroporous sorbent resin was extracted under reduced pressure, washed several times with the appropriate amount of water and collected, extracted 2–3 times with ethyl acetate solvent to remove non-polar impurities, while the aqueous phase was freeze-dried and dissolved in ethanol solution and set aside. Then, the macroporous resin was further eluted with 80% ethanol and the combined ethanol solution was collected and evaporated to obtain 0.296 g DC-TS light yellow powder, the yield was 71.3%.

In order to further characterize and determine the synthesis of DC-TS, ^13^C NMR tests were carried out. ^13^C NMR (400 MHz, CDCl_3_, Figure S1) δ/ppm: 179.27 (C-49, 63), 173.54 (C-1, 7, 32, 33, 61) 37.20 (C-2, 3, 4, 6, 17, 18, 62), 36.41 (C-8, 9, 10, 11, 19, 20, 21, 22), 35.30 (C-13, 42), 34.75 (C-12, 14, 15, 16, 41), 33.22 (C-50), 31.73 (C-28, 29, 30, 31), 29.49 (C-5, 51, 52, 53, 54, 55, 56, 57, 58), 29.47 (C-24, 25, 26), 29.43 (C-23), 29.39 (C-37), 29.33 (C-39, 40), 29.31 (C-47, 48), 29.20 (C-34, 35, 36), 29.17 (C-60), 29.13 (C-27), 25.02 (C-45, 46), 24.96 (C-38), 22.48 (C-59), 13.88 (C-43, 44). HRMS (APCI, *m*/*z*, Figure S2) calculated for DC-TS[M + H]^+^ 1053.27; found: 1053.55.

### 4.3. Preparation of DC-TS Gel

The appropriate amount of DC-TS was uniformly dispersed into the organic solvent in a glass bottle with ultrasonication(KQ-200VDB, 100 W Beijing Xingda Hengxin Technology Co., Ltd., Beijing, China.). Then, the bottle containing DC-TS and organic solvent was put in the water bath at a temperature lower than the boiling point of the solvent and heated for 5 min, and then cooled naturally. If the liquid in the vial stops flowing and becomes solid, it means that the gel has formed. In order to select the gel solvent more accurately, the concentration of forming gel was set as 3 times that in the literature [21], that is, 15 wt%.

### 4.4. Characterization Methods

TS and DC-TS were characterized by Fourier-transform infrared spectrometer (FT-IR). Gels (air-drying at 25 °C room temperature) of DC-TS were characterized by nuclear magnetic resonance spectroscopy (NMR), mass spectrometry (MS), scanning electron microscope (SEM), and X-ray diffractometer (XRD). 

The KBr tableting method was adopted and the test was conducted by FT-IR Bruker TENSOR II in the range of 4000–400 cm^−1^. 

The samples were dissolved in DMSO-d6 and tested for ^1^H NMR spectra at 400 MHz using Germany Bruker Avance III HD NMR (Germany Bruker Company, Karlsruhe, Germany). And DC-TS was dissolved in CDCl_3_-d6 and tested for ^13^C NMR spectra at 400 MHz using Germany Bruker Avance III HD NMR.

The rheological behavior of the polymers was studied at room temperature (25 °C) with a Haake Mars60 rotational rheometer (Haake Technik GmbH, Karlsruhe, Germany) using steel parallel plates. The organogels were prepared in 10 mL centrifuge tubes. Rheology was tested immediately after the organogels’ formation. The organogels were placed on a Haake Mars60 rotational rheometer to study the rheological behavior of the polymers using the steel parallel plate method. Firstly, the stress values should be determined, and after fixing the stress values in the linear viscoelastic region, the elastic modulus G′ and viscous modulus G″ were determined at room temperature at different frequencies. The frequency variation was in the range of 0.1–100 Hz.

The surface structure of the gel was micrographed using an SU-8010 SEM (Hitachi, Ltd., Tokyo, Japan) at an accelerating voltage of 3 KV after the samples were sprayed with gold.

Diffractograms were recorded on a Rigaku Ultima IV XRD (Rigaku Corporation, Tokyo, Japan) with Cu Kα radiation (λ = 1.5406 Å) at a voltage of 45 kV, a current of 100 mA, and a scan speed of 2°/min.

## Figures and Tables

**Table 1:** Gel ability of DC-TS in different solvents and Kamlet–Taft model.

Solvent	Gel Ability		Kamlet–Taft Model		
	Solubility	Gel Capacity	α	β	π*
Petroleum ether (PE)	S	G	0	0	0.10
Cyclohexane (CH)	S	G	0	0	0
N-octane (OT)	S	PG	0	0	0.08
N-heptane (HT)	C	G	0	0	0.08
Carbon tetrachloride (CT)	S	G	0	0	0.28
Toluene (TL)	C	G	0	0.11	0.54
Ether (ET)	I	PG	0	0.47	0.27
Benzene (BZ)	C	G	0	0.10	0.59
Tetrahydrofuran (TD)	C	U	0	0.54	0.51
Ethyl acetate (EA)	I	PG	0	0.45	0.55
Trichloromethane (TM)	C	U	0.44	0	0.58
Acetone (AT)	I	PG	0.08	0.48	0.71

α, the ability of the solvent to donate hydrogen bonds; β, the ability of the solvent to accept hydrogen bonds; π*, generalized polarity parameter; G, gel; PG, partial gel; U, ungel; I, insoluble; S, slightly soluble; C, complete dissolution.

**Table 2:** FT-IR Vibration peaks of DC-TS in different solvents.

	DC-TS	Petroleum Ether	Cyclohexane	N-Heptane	Carbon Tetrachloride	Toluene	Benzene
υ_as_CH_2_	2920	2919	2920	2921	2919	2921	2921
υ_s_CH_2_	2851	2850	2851	2850	2850	2850	2850
υC=O	1719	1721	1718	1717	1718	1719	1717
υC-O-C	1468	1467	1469	1467	1468	1467	1469
δ_as_CH2	1403	1406	1400	1407	1404	1407	1412
δ_s_CH2	1082	1070	1074	1074	1079	1072	1072

## Data Availability

The data presented in this study are openly available in article.

## Supplementary Materials

The following supporting information can be downloaded at: https://www.mdpi.com/article/10.3390/gels10040225/s1, Figure S1: ^13^C NMR of DC-TS; Figure S2: HRMS spectrum of DC-TS.
