# Supplementary material for: Design and Properties of Novel Hydrophobic Natural Tea Saponin and Its Organogels
Source: Gels. 2024 Mar 26;10(4):225. doi: 10.3390/gels10040225 (PMC11049091; doi:10.3390/gels10040225)
Supplement: Supplementary file 1 [file gels-10-00225-s001.zip › gels-2820234-supplementary.pdf]

Supporting Information

Design and Properties of Novel Hydrophobic Natural Tea Saponin and Its Organogels

Maogong Wang [1,†], Liuxin Yan [2,†], Xuying Guo [2], Xinwei Xing [2], Fengqian Liang [2], Chunrui Han [2,*] and Liujun Liu [2]

[1] CNPC Engineering Technology R&D Company Limited, Beijing 102206, China; wangmg_dri@cnpc.com.cn
[2] MOE Engineering Research Center of Forestry Biomass Materials and Energy, Ministry of Education, Beijing Forestry University, Beijing 100083, China; yanliuxin0505@163.com (L.Y.); guoxy@bjfu.edu.cn (X.G.); yi3210319@bjfu.edu.cn (X.X.); liangfengqian7210401@bjfu.edu.cn (F.L.); lux@bjfu.edu.cn (L.L.)
* Correspondence: hanchunrui@bjfu.edu.cn
† These authors contributed equally to this work and share first authorship.

The supporting information includes 2 figures.
Contents:
Figure S1. ^{13}C NMR of DC-TS
Figure S2. HRMS spectrum of DC-TS.

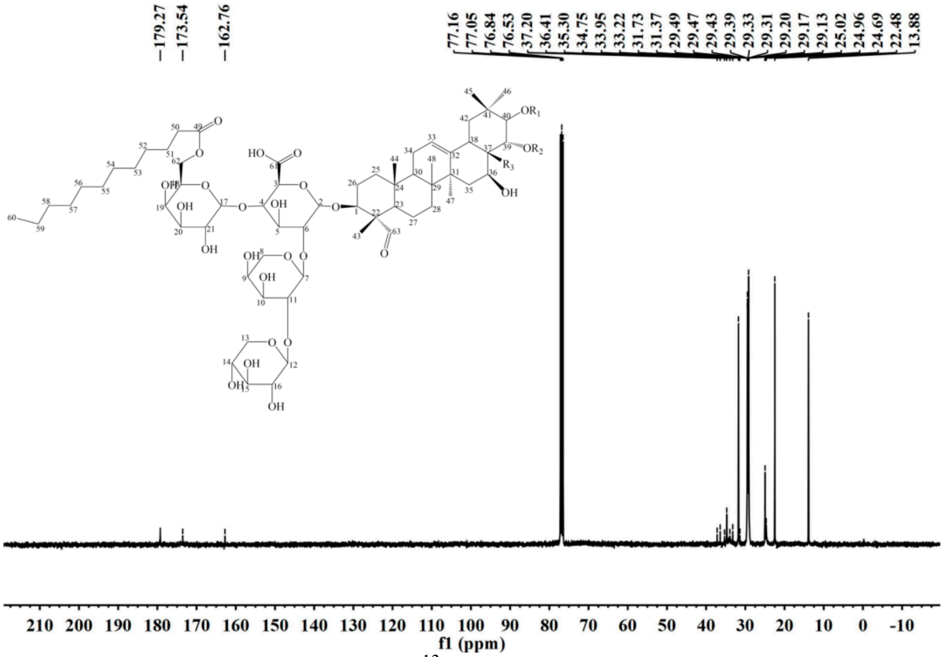

Figure S1. ^{13}C NMR of DC-TS

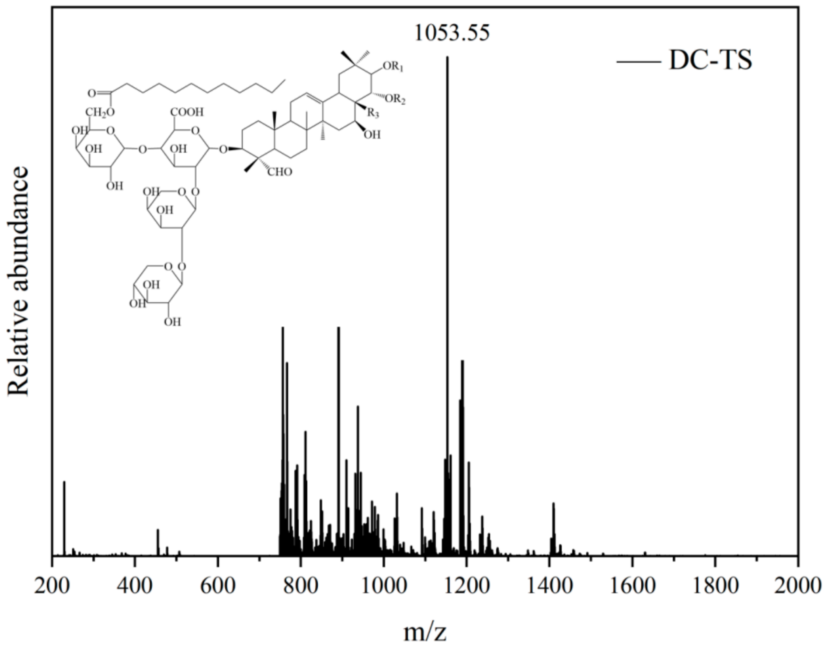

Figure S2. HRMS spectrum of DC-TS.
